# Physical sporting activity impact on long-term BMI trajectories in Dutch adults: a longitudinal observational study

**DOI:** 10.1186/s13690-025-01773-3

**Published:** 2025-12-01

**Authors:** Dirk Lubbe, Stephan Gretschel, Philipp Otto, Ulf Elbelt

**Affiliations:** 1 Institute for Psychological Methods, Department of Psychology, Brandenburg Medical School Theodor Fontane, Am Alten Gymnasium 1-3, Neuruppin, 16816 Germany; 2 Faculty of Health Science Brandenburg, joint faculty of Brandenburg, University of Technology Cottbus-Senftenberg, Brandenburg Medical School Theodor Fontane and University of Potsdam, Neuruppin, Rüdersdorf, Germany; 3 Department of General, Visceral, Thoracic and Vascular Surgery, University Hospital Ruppin-Brandenburg (ukrb), Brandenburg Medical School Theodor Fontane, Neuruppin, Germany; 4 Section Endocrinology/Diabetology, Medical Clinic B, University Hospital Ruppin- Brandenburg (ukrb), Brandenburg Medical School Theodor Fontane, Neuruppin, Germany; 5 MVZ Endokrinologikum Berlin, Berlin, Germany

**Keywords:** Metabolic equivalent task (MET), Physical intensity, Sporting activity, Obesity, Longitudinal measure, Body mass index trajectories, Body weight stability

## Abstract

**Background:**

Sporting activity is regarded as one of the key determinants of energy expenditure and is often recommended as a part of weight management. However, on a population basis the general long-term impact of initiating or modifying sporting activities on body mass index (BMI) remains insufficiently understood. This longitudinal study examines the relationship between sporting activity intensity, measured via metabolic equivalent task scores (MET), and BMI trajectories over a five-year period in a representative Dutch sample. Moderating effects of age, gender, and baseline BMI were assessed to identify population subgroups with differential responsiveness.

**Methods:**

Data were drawn from the Longitudinal Internet Studies for the Social Sciences (LISS) panel (*N* = 6,337), which provided annual self-reported measures of BMI and sporting activities between 2008 and 2023. At baseline, the average age of participants was 47.1 years (sd = 17.0; median = 48.0) and 54.0% were female. Sporting activity intensity was operationalized using MET scores assigned to reported sport types. Linear mixed-effects models were used to evaluate the association between within-person changes in MET and BMI trajectories, adjusting for age, gender, and baseline BMI category. Interaction terms tested whether associations varied by obesity category and age group.

**Results:**

Cross-sectional analyses indicated that MET explained approximately 2% of the variance in BMI. Longitudinally, annual changes in sporting activity intensity were associated with only minimal but significant shifts in BMI for most participants. However, relevant effects emerged only in individuals with obesity (BMI ≥ 30). Specifically, starting to engage in a sporting activity with a MET rating of about 5 (such as moderate cycling) would be associated with an average annual BMI reduction of 0.3 points in individuals with class I obesity and 0.5 points for class II obesity. Among participants without obesity or those aged ≥ 65 years, BMI changes associated with MET variation were negligible. Gender did not significantly moderate the relationship.

**Conclusions:**

In this large population-based sample from the Netherlands, sporting activity intensity showed a statistically significant but overall small impact on BMI trajectories, with the largest benefits concentrated in younger adults and individuals with obesity. These findings stress the importance of early and sustained engagement in higher-intensity sporting activities for weight management, while also pointing to the need for comprehensive strategies—including dietary interventions and medical options—for individuals with severe obesity or advanced age for whom high intensity sporting activities are not appropriate. Clinicians should provide realistic counselling about the expected magnitude of BMI change from sports alone and tailor recommendations to patients’ weight status and functional capacity.

**Table:** 

Text box 1. Contributions to the literature
• Further insight into longitudinal dependencies between intensity of sporting activities and body weight development.
• Effects of changes in sporting activity depend on baseline body mass index levels and age.
• Clinicians' recommendations should take into account the limited effect of increased sporting activity on weight development and convey realistic expectations regarding the impact of increased exercise in health education.

## Background

Overweight and obesity are among the leading drivers of global morbidity and mortality [1], with prevalence continuing to rise across high- and middle-income countries, underscoring the urgent need for effective prevention and management strategies [2, 3]. Body mass index (BMI) remains the most widely used surveillance indicator, though it only partially reflects body composition and metabolic health risk [4]. Tracking changes in BMI over time can nevertheless provide valuable insights into the dynamics of weight management at the population level [5, 6].

Physical activity is a central modifiable determinant of energy expenditure and therefore is thought to play a crucial role in weight management and prevention of weight gain over time [7]. Its positive effects extend beyond weight control, impacting cardiovascular fitness, mental health, and metabolic markers [8]. The intensity of physical activity is commonly expressed as metabolic equivalent of task (MET), which quantifies the energy expenditure of various physical activities relative to resting metabolism [9]. Sporting physical activities (SPA) in particular often reach higher MET levels than everyday lifestyle activity, making them a key candidate for weight-management interventions [10].

Although numerous observational studies report inverse associations between SPA and BMI [11], the strength of this relationship is modest, typically accounting for only a small fraction of weight loss over time [12]. These results are in line with experimental studies, which report moderate effects (Cohen’s d ≈ − 0.50 or analogously 6% of explained variance) of exercise-only interventions [13, 14].

However, effects pertaining to cross-sectional observational or experimental studies do not necessarily reflect long-term changes in representative populations under real-world conditions. Only few studies have examined whether within-person changes in sport-related activity intensity predict BMI trajectories across multiple years [15]. Moreover, clinical trials, while providing strong causal inference, are conducted in highly controlled environments, often with motivated participants and structured protocols that do not mirror everyday life. Similarly, cross-sectional observational studies capture associations at a single point in time, limiting insight into dynamic processes and potential reverse causality.

Therefore, analyses based on large, representative longitudinal samples may provide a helpful additional perspective on how changes in SPA influence BMI under naturalistic conditions. Examining within-person changes across time allows for ecologically valid estimates of the effect of SPA on weight trajectories, while accounting for demographic heterogeneity. Importantly, the meaningful analysis of heterogeneous effects across sub-populations—such as those defined by age, gender, or baseline BMI—depends on the representativeness of the sample to ensure that findings are generalizable to the broader population. In this context, several moderating factors warrant attention [15].

Gender differences influence both the type and intensity of SPA [16–19]. Men typically have a higher lean body mass and resting metabolic rate, leading to greater energy expenditure during physical activity compared to women [20]. They are also more likely to engage in high-intensity, high-MET activities, whereas women often prefer moderate-intensity exercise and may face social or structural barriers to vigorous sports [21, 22].

Age is another critical moderator. Physiological changes with aging, such as declining muscle mass and metabolic rate, reduce energy expenditure and limit participation in high-MET activities [23, 24]. Older adults often shift towards lower-intensity exercise, and age-related health constraints further diminish potential BMI reductions from sporting activity [25].

Baseline BMI also shapes activity patterns. Individuals with obesity encounter physical limitations that restrict engaging in high-intensity or long-duration activities, leading to engagement in lower-MET activities with smaller weight-loss effects [26, 27]. Conversely, individuals with lower BMI can adopt high-intensity sports more easily, sustaining more pronounced energy expenditure benefits [12].

These demographic and physiological factors highlight the need for stratified analyses that differentiate the expected impact of SPA by gender, age, and initial weight status - an approach central to the present study.

Against this background, the present study aims to provide population-based evidence on the longitudinal relationship between SPA intensity and BMI trajectories under naturalistic conditions. Using data from a large, nationally representative Dutch cohort with repeated annual assessments, we analyze how both the level and changes in SPA, expressed via MET, relate to changes in BMI over a five-year period [28]. A key objective is to examine whether these associations differ by age, gender, and baseline BMI category, thereby identifying subgroups with greater or lesser responsiveness. On a practical level, individuals often overestimate the impact of increased SPA on weight regulation and may become discouraged when temporary increases in activity do not lead to substantial weight loss [26, 29]. This study therefore aims to contribute to a more differentiated understanding of the effects of increased SPA in order to be able to convey realistic expectations during consultations.

## Methods

### Study population

This study utilized data from the Longitudinal Internet Studies for the Social Sciences (LISS) panel, a publicly available, ongoing longitudinal panel in the Netherlands. Established in 2007 and managed by CentERdata at Tilburg University, the LISS panel annually collects information on a wide range of topics, including social, economic, and demographic factors, as well as data on health, well-being, and lifestyle behavior. Participants were randomly drawn from the Dutch population registers and invited to participate. The initial sample in 2007 included 8,000 participants from almost 5,000 households. Ongoing recruitment of new participants compensates for attrition and helps maintain panel size. Detailed information about the study design and access to the datasets is available on the LISS data archive (dataarchive.lissdata.nl).

From the overall LISS sample, our group identified all participants who had provided at least five annual weight assessments (spanning at least five years). These repeated measures were necessary for reliable estimation of individual BMI trajectories over time. Participants were excluded if they had a baseline BMI below 18.5 (underweight) or if their reported heights and weights were implausible. Specifically, values below 1.30 m or above 2.50 m for height and below 30 kg or above 250 kg for weight were excluded. In total, 6337 participants were identified as viable cases for data analysis.

### Ethical considerations

All participants in the LISS panel provided informed consent in accordance with Dutch legal and ethical guidelines. The panel data are anonymized before public release to protect participant confidentiality. Ethical review for secondary analyses of anonymized data typically is not required under Dutch law; however, researchers using these data must comply with institutional and national data protection regulations.

### Variables and measurements

The main outcome variable for the confirmatory analysis was the change of BMI from the baseline assessment. BMI values were calculated based on the annual self-reported values of height (in m) and weight (in kg) by dividing body weight (kg) by the square of height (m^2^).

As measures of SPA, we included (a) reports of general sporting activity engagement, which included an annual binary (yes/no) response whether participants regularly engaged in any sporting activity and, if so, the weekly hours spent doing so, (b) binary indicators that captured the specific types of sports practiced (a list of the assessed sports can be found in Table 2), as well as (c) a metabolic equivalent of task (MET) value that was estimated based on the reported sporting activities. Specifically, each reported sport was assigned a standard MET value reflecting its average energy cost relative to resting metabolism using the categorization by Herrmann et al. [30], which is displayed in Table 2. For participants engaging in multiple sports, an average MET score was computed by summing each activity’s MET value and dividing by the total number of sports reported.

As demographic and clinical covariates, we included age, gender, and the BMI category at baseline, which subdivides participants with normal weight (BMI between 18.5 and 24.9), overweight (BMI between 25.0 and 29.9), class 1 obesity (BMI from 30.0 to 34.9) and higher-class obesity (BMI of 35 or higher).

Measurements of these variables were collected throughout the year and are contained in different core studies of the LISS panel. Specifically, BMI was assessed annually in November and December, whereas variables pertaining to sports were either assessed from February to March in the years 2008 to 2014 or from October to November in the years 2015 to 2023 and matched by individual identifiers.

### Statistical analyses

All analyses were performed in R version 4.4.1 (R Core Team, Vienna, Austria). Statistical significance was set at *p* <.05 unless stated otherwise.

Baseline participant characteristics, including age, gender distribution, and BMI are summarized using descriptive statics. Specifically, we report means, standard deviations and quantiles for the metric variables age, MET, and hours of sports per week stratified by gender and BMI category as well as frequencies and percentages of reported sporting activities stratified by BMI category. Moreover, the sample’s BMI distribution and the product-moment correlations among the metric variables are considered. Furthermore, we conducted an exploratory multiple regression analysis in which baseline BMI was regressed on binary indicators representing participation in individual sporting activities. This analysis served as a plausibility check to assess whether the regression coefficients for different types of sporting activities were consistent with their expected effects on BMI. Beyond this purpose, further interpretation of the coefficients should be approached with caution, as some individuals reported engaging in multiple sporting activities, and all activity indicators were included as additive terms, which may not fully capture the true underlying relationships.

As descriptive statistics for the longitudinal relations, we provide mean plots with 95% confidence intervals for changes in BMI and MET relative to baseline stratified by age and BMI category.

Confirmatory, longitudinal data analysis was performed using linear mixed-effects models with restricted maximum likelihood (REML) estimation to account for within-subject correlations over repeated measurements. The primary outcome variable was the annual difference in BMI ($\:{\Delta\:}\text{B}\text{M}\text{I}$) from each individual baseline value. We included the following independent variables as fixed effects:

- time of measurement ($\:t$): modelled as a polynomial spline of the second degree to capture potential non-linear BMI changes over time.
- age: four-level ordinal variable (1: up to 35 yrs; 2: over 35 to 50 yrs; over 50 to 65 yrs; over 65 yrs)^1^
- gender: female or male.
- average MET across timepoints ($\:\stackrel{-}{\text{M}\text{E}\text{T}}$): The mean MET across all timepoints for each participant, reflecting typical activity intensity.
- annual difference from the personal MET average ($\:{\Delta\:}\text{M}\text{E}\text{T}$): The difference between the participant’s annual MET and their own average MET, this captures year-to-year fluctuations around each individual’s typical level of activity intensity.
- the participants BMI-category ($\:{\text{B}\text{M}\text{I}}_{c}$): four-level ordinal variable (1: BMI 18.5 to 24.9 kg/m²; 2: BMI 25.0 to 29.9 kg/m²; 3: BMI 30.0 to 34.9 kg/m²; 4: BMI 35.0 kg/m² or higher).

As random effects we included:

- individual random intercepts.
- random slopes of $\:t$.

Starting with a model that included all listed variables, including their three-fold interactions, we removed all effects that did not significantly contribute to explaining changes in BMI using a stepwise elimination algorithm. Specifically, this algorithm starts with the highest order interactions and iteratively removes all effects that do not reach significance at a 5% level.

### Missing values

Prior to modelling, missing data patterns were assessed. Specifically, we analyzed whether missing values may be considered *missing completely at random* (MCAR) using Little’s MCAR-Test. If missing patterns do not deviate strongly from the MCAR assumption, they should have a negligible effect on the parameter estimates.

Overall, 9.7% of potential data patterns of the longitudinal data were missing, which is an acceptable amount. Although Little’s MCAR reaches statistical significance ($\:{\chi\:}^{2}=3352.07$ $\:\text{d}\text{f}=2317$, $\:p<.001$), the effect of missingness may be considered as very small. This may be concluded from the test statistic’s RMSEA (root mean square error of approximation) which reaches a value of 0.0084. Given the relatively minor degree of missingness and the small effect size implied by the RMSEA, we proceeded with a complete-case analysis in the mixed-model framework.

## Results

### Baseline descriptive analyses

**Fig. 1:**
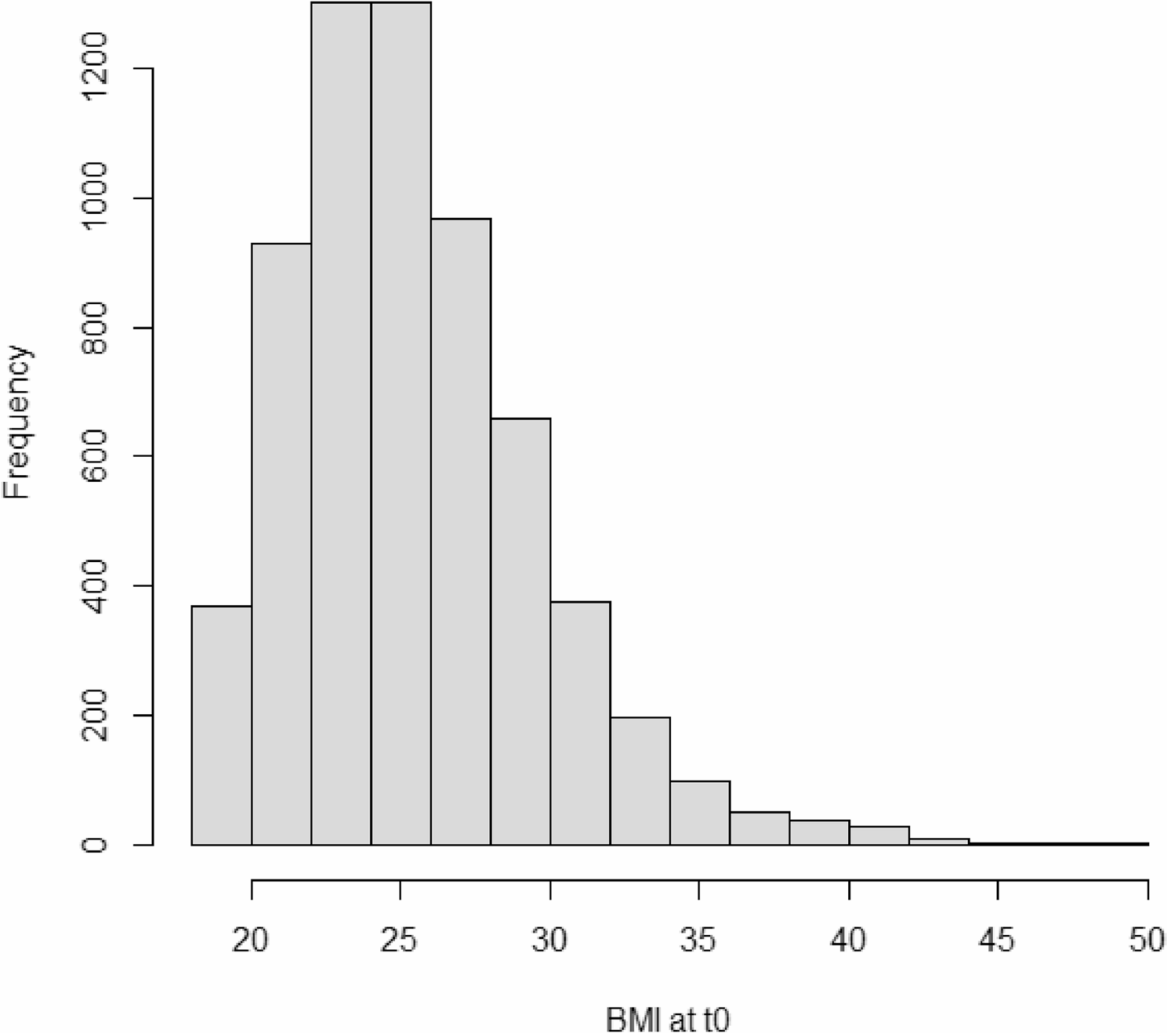
Histogram of BMI values at baseline

The total data set includes 6337 participants, of which 3422 (54.0%) were female. The mean age of participants was 47.1 years (sd = 17.0; median = 48.0). A total of 3324 (52.4%) participants had a BMI below 25 kg/m², 2202 (34.7%) had a BMI between 25 and 29.9 kg/m², 629 (9.9%) had a BMI between 30 and 34.9 kg/m², and the remaining 182 participants reported values of or above 35 kg/m² (2.9%). The distribution of BMI at baseline is displayed in Fig. 1.

At baseline, 5377 (84.9%) participants of the total sample provided information on their individual sporting activities. Of these participants, 3088 (57.4%) participants reported regularly engaging in sporting activities. The average MET, including participants that reported not engaging in sporting activities, was 3.1 (sd = 2.9; median = 4.0) and the average hours of sport per week were 2.4 (sd = 3.5; median = 1.5), both values declining with increasing weight category. Table 1 gives average values of age, MET, and hours of sports per week by gender and BMI category.

**Table 1:** Descriptive statistics of study variables by gender and BMI-category

	Gender	BMI	*N* (%)	Mean	SD	Q1	Median	Q3
Age	Male	18.5–24.9.5.9	1384 (21.8)	44.48	18.27	29.0	45.0	60.0
		25.0–29.9.0.9	1194 (18.8)	51.83	14.44	41.0	53.0	63.0
		30.0–34.9.0.9	289 (4.6)	53.38	13.04	44.0	54.0	63.0
		35.0	48 (0.8)	52.31	12.75	45.8	52.0	61.0
	Female	18.5–24.9.5.9	1940 (30.6)	42.22	17.57	27.0	42.0	56.0
		25.0–29.9.0.9	1008 (15.9)	51.13	15.23	41.0	53.0	62.0
		30.0–34.9.0.9	340 (5.4)	49.96	15.44	38.0	50.0	61.0
		35.0	134 (2.1)	48.34	14.60	37.0	49.0	60.8
MET	Male	18.5–24.9.5.9	1171 (18.5)	3.64	3.21	0.0	4.5	5.9
		25.0–29.9.0.9	1032 (16.3)	3.05	2.93	0.0	4.0	5.0
		30.0–34.9.0.9	250 (3.9)	2.38	2.72	0.0	0.0	4.8
		35.0	39 (0.6)	2.19	2.65	0.0	0.0	4.8
	Female	18.5–24.9.5.9	1612 (25.4)	3.29	2.84	0.0	4.0	5.0
		25.0–29.9.0.9	859 (13.6)	2.69	2.65	0.0	3.7	5.0
		30.0–34.9.0.9	299 (4.7)	2.30	2.58	0.0	0.0	4.6
		35.0	115 (1.8)	1.87	2.43	0.0	0.0	4.3
Sport	Male	18.5–24.9.5.9	1171 (18.5)	3.02	3.96	0.0	2.0	5.0
h/w		25.0–29.9.0.9	1032 (16.3)	2.45	3.31	0.0	2.0	4.0
		30.0–34.9.0.9	250 (3.9)	2.11	3.31	0.0	0.0	3.0
		35.0	39 (0.6)	1.90	2.67	0.0	0.0	3.5
	Female	18.5–24.9.5.9	1612 (25.4)	2.36	3.37	0.0	2.0	3.0
		25.0–29.9.0.9	859 (13.6)	2.03	3.26	0.0	1.0	3.0
		30.0–34.9.0.9	299 (4.7)	1.82	3.50	0.0	0.0	3.0
		35.0	115 (1.8)	1.10	1.60	0.0	0.0	2.0

*%* percentage of the total sample (*n*=6337), Q1: 25% percentile, Q3: 75% percentile, Sport h/w: self-assess hours of sporting activities per week

The percentage of participants engaging in different types of sport is displayed in Table 2. Results are given for the total sample as well as separated by BMI-category. All types of sport, except swimming and gymnastics, were reported more often in the lower weight categories. This difference was most pronounced for running, a sporting activity with the highest MET.

**Table 2:** Type of sport, MET scores and frequency of engaging in sporting activities for 5377 participating adults at baseline

Type of sport	MET	%	% by weight category			
			18.5–24.9.5.9	25.0–29.9.0.9	30.0–34.9.0.9	35.0
Running	10.5	10.3	13.8	7.8	3.8	1.9
Soccer	8	4.1	5.2	3.3	2.4	0.0
Hockey	8	0.9	1.0	0.7	0.7	0.6
Inline skating	7	1.3	2.0	0.7	0.4	0.0
Swimming	6	8.5	8.2	8.9	8.2	9.1
Dancing	6	3.2	4.7	2.0	1.6	0.0
Cycling	5.5	17.6	19.1	18.0	10.2	9.7
Gymnastics	5	3.5	4.1	3.0	2.4	4.5
Fitness	4	20.9	21.1	21.0	20.0	18.8
Golf	3.5	1.6	1.4	2.1	1.3	0.6
Walking	3.5	18.2	18.8	19.0	14.0	13.0
Other sport	5	13.9	16.1	11.7	11.1	10.4

Correlations for BMI values and three different sport indicator variables at baseline are shown in Table 3. All correlations reach statistical significance at an alpha-level of 0.001. BMI yields the strongest correlation with the MET and MET h/w (for both $\:-.15$), denoting that MET and MET h/w explain about 2% of the overall BMI variance. However, the reported hours of sports per week do not contribute additional information when used as weight for the MET score. Therefore, in all further analyses only the MET is included.

**Table 3:** Pearson correlations of BMI values and sport indicators at baseline

	(1) BMI	(2) MET-score	(3) Sports h/w	(4) MET h/w
(1) BMI	1.00			
(2) MET-score	−0.15***	1.00		
(3) Sports h/w	−0.09***	0.53***	1.00	
(4) METh/w	−0.15***	0.89***	0.79***	1.00

Variable (4) METh/w is the product of variables (2) MET-score and (3) hours of sport per week, *h/w* hours/week

To explore the association of individual types of sport with the BMI, we calculated a multiple regression of individual sport choices (based on the binary indicator variables) on BMI values at baseline. While gender and age account for about 4 to 5% of the BMI variance, whereby higher BMI values are connected to older age and male gender, the individual sport preferences account for additional 2% of the BMI variance. The regression coefficients for the individual sporting activities are given in Table 4. Largest negative regression coefficients result for running and inline skating. In particular, the regression coefficient pertaining to running of −1.15 indicates that individuals who run have on average 1.15 BMI-points less than other individuals with the same age and gender, whereas for individuals who perform inline skating, BMI-values are comparably decreased by 0.95 points. There are also considerable differences in BMI depending on whether participants reported cycling, gymnastics, and soccer, which indicate lower BMI values by 0.58 to 0.68 points. Interestingly, for participants who swim, an *increased* BMI by 0.51 points is predicted. Note that the size of regression coefficients is in a close linear relation with the sports’ MET.

**Table 4:** Linear regression coefficients of age, gender, and different types of sport predicting BMI values at baseline

	Coefficient	95 %-CI	*P*
[Intercept]	23.87	23.33,24.40	<0.001***
Age	0.05	0.05, 0.06	<0.001***
Gender[female]	−0.39	−0.62,−0.17	<0.001***
Running	−1.15	−1.52,−0.78	<0.001***
Soccer	−0.57	−1.14,−0.01	0.047*
Hockey	−0.22	−1.39, 0.94	0.708
Inline skating	−0.95	−1.91, 0.01	0.051
Swimming	0.51	0.11, 0.91	0.012*
Gymnastics	−0.68	−1.28,−0.08	0.026*
Cycling	−0.68	−1.00,−0.35	<0.001***
Fitness	0.26	−0.01, 0.53	0.061
Golf	−0.30	−1.16, 0.56	0.499
Walking	−0.32	−0.64, 0.01	0.056
Other sport	−0.43	−0.74,−0.11	0.008**

### Longitudinal descriptive analyses

Next, a selection of longitudinal relations among these variables is displayed descriptively using mean plots to give a more comprehensive overview of the relations within the data. First, we consider changes in BMI over time ($\:{\Delta\:}\text{B}\text{M}\text{I}$) for separate average BMI levels and age groups. Second, BMI changes are considered in connection with annual changes in MET ($\:{\Delta\:}\text{M}\text{E}\text{T}$). Third, changes in MET over time are plotted for separate average BMI levels and age groups.

**Fig. 2:**
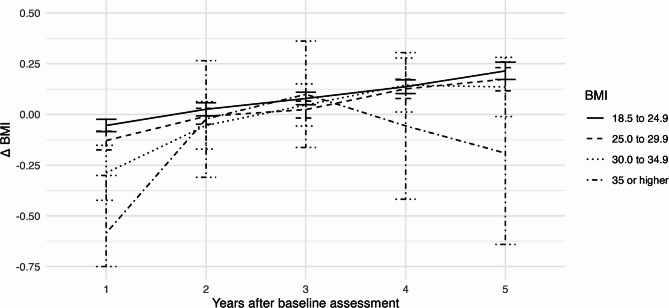
Mean values with 95% confidence intervals of BMI changes ($\:{\Delta\:}\text{BMI}$) from baseline over time by initial BMI value

BMI changes over time for different weight categories are shown in Fig. 2. In general, BMI values increase over time, whereby the annual increase is on average only roughly 0.10 BMI points. This trend fits average BMI changes in individuals up to a BMI of 35 very well. However, for participants with an average BMI of 35 or higher, which is the smallest subgroup, the trends are not as uniformly shaped, which may be partially attributed to the lower precision of mean estimates, as indicated by the large confidence intervals.

**Fig. 3:**
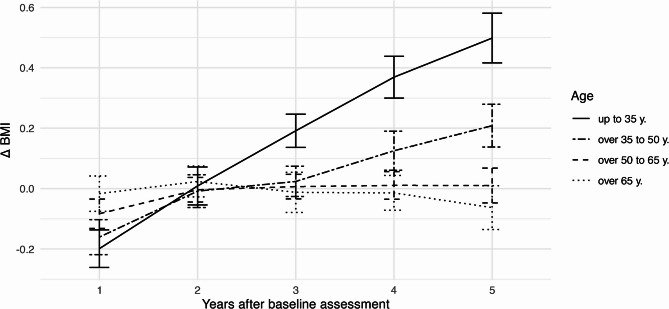
Mean values with 95% confidence intervals of BMI changes ($\:{\Delta\:}\text{BMI}$) from baseline over time by age

BMI changes over time for the four age groups are displayed in Fig. 3. Annual increases in participants up to 35 years old are strongest, averagely increasing by 0.15 BMI points. In age groups over 65 years, there is no systematic increase in BMI values over time.

**Fig. 4:**
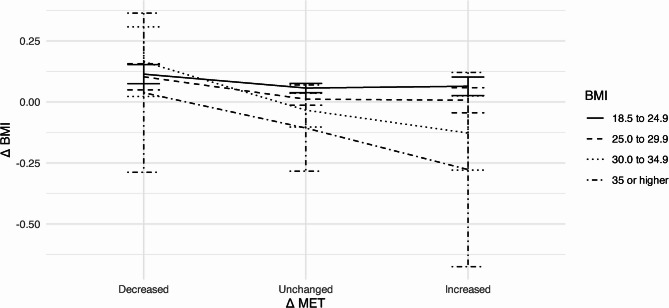
Mean values with 95% confidence intervals of BMI changes ($\:{\Delta\:}\text{BMI}$) from baseline depending on changes in MET ($\:{\Delta\:}\text{MET}$) by initial BMI value; *decrease*: change in MET ≤ −2; *increase*: change in MET ≥ 2; *unchanged*: change in MET < |±2|

Changes in BMI depending on an increase or decrease of MET by weight category are shown in Fig. 4. Interestingly, changes in MET in participants with obesity (BMI ≥ 30) descriptively correspond with a stronger effect on BMI than for participants without obesity.

**Fig. 5:**
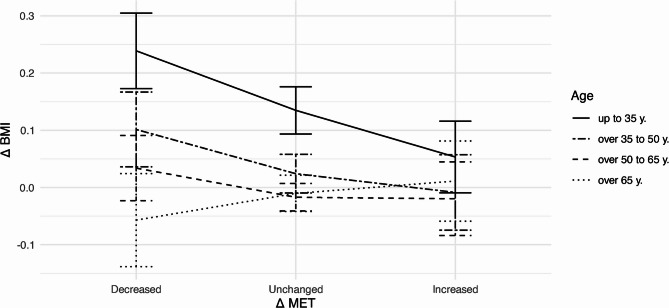
Mean values with 95% confidence intervals of BMI changes ($\:{\Delta\:}\text{BMI}$) from baseline depending on changes in MET ($\:{\Delta\:}\text{MET}$) by age; *decrease*: change in MET ≤ −2; *increase*: change in MET ≥ 2; *unchanged*: change in MET < |±2|

Respectively, changes of BMI depending on an increase or decrease of MET by age groups are shown in Fig. 5. Clearly, for younger participants of up to 35 years, BMI values change more strongly with MET changes than for older participants. For participants over 50 years of age, changes in BMI depending on MET are consistently close to zero.

**Fig. 6:**
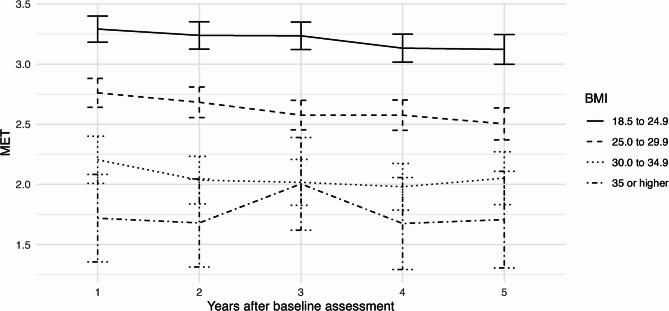
Mean values with 95% confidence intervals of MET over time by initial BMI value

Changes of MET over time by initial weight category are illustrated in Fig. 6. The level of MET is distinctly connected with the BMI category, with participants with normal weight having the largest average MET with over 3.5. Notably, in individuals without obesity, there is a small, but continuous decrease in MET over time, which does not hold true for individuals with obesity. However, this lack of a trend may be due to a floor effect or it the lower precision of estimates due to the smaller subsamples.

**Fig. 7:**
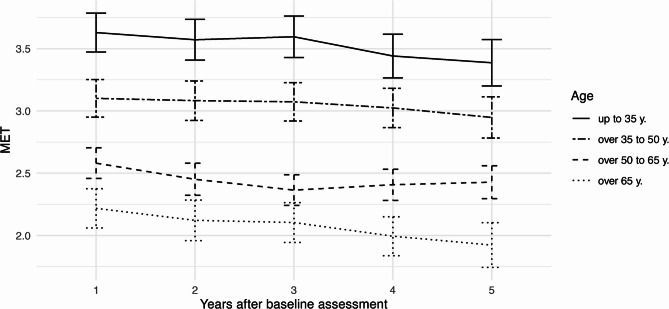
Mean values with 95% confidence intervals of MET over time by age

Finally, the change of MET over time by age is displayed in Fig. 7. Clearly, the mean level of average MET differs between age groups, whereby younger participants report higher MET on average. Over time, MET appear to be stable across age groups with a minor trend towards decreasing values which appears to be somewhat stronger in participants in the highest age category.

### Linear mixed model results

In the following we present the results of the linear mixed model that tests the specific effects of time of measurement ($\:t$), age, gender, average MET across timepoints ($\:\stackrel{-}{\text{M}\text{E}\text{T}}$), annual difference from $\:\stackrel{-}{\text{M}\text{E}\text{T}}$ ($\:{\Delta\:}\text{M}\text{E}\text{T}$), and initial weight-category ($\:{\text{B}\text{M}\text{I}}_{c}$) on changes in BMI. After the backwards model selection process, gender and $\:\stackrel{-}{\text{M}\text{E}\text{T}}$ were removed from the model, because they did not contribute incrementally to explaining BMI changes beyond the other variables.

The terms of the fixed effects of the final linear mixed model are given in Table 5 together with their significance tests and effect-size measures (part. $\:{\eta\:}^{2}$: partialized eta-squared). Only time of measurement ($\:t$), age, the initial weight-category ($\:{\text{B}\text{M}\text{I}}_{c}$), and the annual MET change ($\:{\Delta\:}\text{M}\text{E}\text{T}$) remain as independent variables. While almost all model terms reach statistical significance, effects sizes are consistently small, explaining only up to 1% of the BMI-changes respectively. Only $\:t$ explains approximately 2% ($\:{\eta\:}^{2}=.0200$) of the systematic changes in BMI, characterizing a continuous, but small increase in BMI over time, matching the result of the descriptive analyses.

**Table 5:** ANOVA table pertaining to the fixed effects for predicting changes in BMI Values. Significance testing was performed using satterthwaite adjusted degrees of freedom

Variable	$F$	$df_{num}$	$df^\ast{{}}_{den}$	$p$	part.$n^2$
Time ($\:t$)	60.24	2	5609.0	< 0.001 ***	0.0200
Age	6.36	3	6113.0	< 0.001 ***	0.0031
BMI category ($\:{\text{BMI}}_{c}\text{}$)	5.13	3	6101.3	0.002 **	0.0025
MET change ($\:{\Delta\:}$MET)	25.09	1	21089.8	< 0.001 ***	0.0012
$\:t\:$* Age	12.81	6	5579.3	< 0.001 ***	0.0100
$$\:t*$$$$\:{\text{BMI}}_{c}$$	8.90	6	5571.3	< 0.001 ***	0.0095
Age *$\:{\text{BMI}}_{c}$	0.96	9	6095.7	0.468 (n.s.)	0.0014
$$\:{\text{BMI}}_{c}*$$$$\:{\Delta\:}$$MET	5.20	3	21179.2	0.001 **	0.0007
$\:t\:\text{*}\:\text{A}\text{g}\text{e}\:\text{*}\:{\text{BMI}}_{c}$	2.23	18	5542.4	0.002 **	0.0072

Notes.$\:{\text{d}\text{f}}_{den}^{*}$were calculated with Satterthwaite’s method; the partial $\:{\eta\:}^{2}$ gives the proportion of overall variance that is explained by the individual terms

MET change ($\:{\Delta\:}$MET) has a significant main effect on BMI changes, yielding a small effect size of $\:{\eta\:}^{2}=.0012$. The size of this effect varies between weight groups, as indicated by the significant $\:({\text{BMI}}_{c}*{\Delta\:}\text{M}\text{E}\text{T}$)-interaction with a small effect size of $\:{\eta\:}^{2}=.0007$, which indicates stronger effects of subpopulations with higher BMI. Specifically, the effect of $\:{\Delta\:}$MET in participants with differing baseline BMI can be studied more closely by considering the corresponding model estimates. For changing sporting behavior towards an activity with one additional MET-point, an annual change of − 0.061 BMI points is expected on average for participants with a BMI between 30 and 34.9 kg/m². The effect is even stronger for individuals with a BMI of 35 kg/m² or above with an expected change of − 0.100 kg/m² per additional MET. For participants with normal wight, changes in MET have the smallest effect, yielding a coefficient of − 0.031 kg/m², followed by a similarly small effect of individuals with overweight of − 0.037 kg/m².

The remaining three-fold interaction ($\:t$ * age * $\:{\text{BMI}}_{c}$) indicates that the change of BMI values over time is different for distinct combinations of age and initial weight category. Specifically, participants of up to 35 years of age report the strongest annual increases in BMI if their initial BMI was 35 kg/m² or higher. For participants between over 35 and 45 years of age, the annual increases in BMI are particularly strong if the initial BMI was 35 kg/m² or higher. For older participants, there is no notable difference in annual BMI changes depending on their initial BMI.

To conclude, we report the random effects of the model. The random intercept has a variance of 1.156 (95% CI = 1.077, 1.228), the random slopes of $\:t$ have a variance of 2.129 (95% CI = 2.054, 2.370) for the linear part and 2.381 (95% CI = 2.214, 2.532) for the quadratic part, and the residual variance is 0.925 (95% CI = 0.900, 0.952). Linear and quadratic random slopes are moderately correlated with a coefficient of 0.58. These effects illustrate that there are substantial differences in individual trajectories, which cannot be explained by the investigated variables.

## Discussion

This study examined the longitudinal relationship between self-reported sporting activities and BMI levels in a large, representative Dutch sample. By focusing on annual changes in MET over five years, we aimed to quantify the impact of sporting physical activity (SPA) on weight changes and identify central factors affecting weight trajectories. While our findings replicate and confirm known relations between sporting activities and body weight, we provide additional insights in the differential effects of distinct subpopulations as well as directed effect of SPA on BMI in contrast to mere correlational estimates under largely representative circumstances.

At baseline, less than half of the individuals (48.7%) of this representative sample reported to engage in sporting activities. Notably, the median MET and the median hours of sport per week of participants with obesity were zero respectively. Individuals with high BMI prefer sports with low- to medium MET, such as swimming and gymnastics. Lower BMI values were associated with higher MET and engagement in vigorous activities such as running or inline skating. These findings are in line with previous reports on exercise-related activity thermogenesis that showed to be almost negligible in individuals with obesity [11]. Notably, participants reporting running exhibited a 1.15-point lower BMI compared to non-runners, independent of age and gender, aligning with the high MET rating of this activity. Conversely, swimming was linked to a slight BMI increase (+ 0.51 points) a finding that is consistent with our clinical experience that people with obesity prefer swimming as their form of sporting activity, but requires further examination in comparison to not-swimming.

Cross-sectionally, MET explained approximately 2% of BMI variance, highlighting SPA as a modest but consistent correlate of weight status. Clearly, the fact that we measure MET based on the average MET of broad categories of sporting activities – no differentiation between low and high intensity performance within individual categories – adds randomness to our analyses. Thus, the amount of explained variance is likely to be larger if more specific MET values would have been available. In addition, the fact that weight and sporting activities were assessed at different timepoints within a year may also lead to somewhat decrease measures of association as compared to a simultaneous assessment. Nevertheless, the small explained variance also underscores the multifactorial nature of BMI regulation, where factors beyond exercise - such as nutritional energy intake, genetics, and metabolic parameters (e.g. insulin resistance) - likely play more substantial roles.

The longitudinal BMI value analyses showed a small but consistent general annual BMI increase (+ 0.1 points). This trend was strongest in individuals up to 35 years of age (up to + 0.15 points/year), whereas a small decline was observed for individuals above 65 years of age. These findings are consistent with the literature [31].

Annual changes in SPA related MET, on the other hand, contributed only marginally to BMI changes. Although the corresponding main effect reached statistical significance, its effect size (part. $\:{\eta\:}^{2}$ = 0.0012) is very small. However, subgroup analyses showed clinically meaningful patterns depending on the BMI level at baseline. Specifically, participants with obesity (BMI ≥ 30) demonstrated greater BMI reductions in response to engaging in a sporting activity with higher MET, with a one point increase in MET predicting declines of 0.061–0.100 BMI points depending on the degree of baseline obesity. The descriptive analysis furthermore indicates that younger individuals (< 35 years) also exhibited stronger BMI responsiveness to MET changes compared to older adults, whose weight trajectories remained largely unaffected by activity shifts. However, the corresponding term was not included in our final linear mixed-effects model due to lack of statistical significance. Gender did not significantly alter longitudinal BMI trajectories once these factors were taken into account.

The divergence between longitudinal and cross-sectional effect sizes underscores that a substantial part of the overall association between SPA and BMI cannot be attributed to a direct effect from the former on the latter. A substantial part of the effect may be either inversely related (for instance because individuals of lower BMI are more inclined to engage in higher-intensity sports) or caused by other biological, socio-behavioral, or economic variables. While it is difficult to derive an estimate of the expected contribution of directed versus inverse causation, because we have no information on (retest-) reliability of the self-reported measures, the overall discrepancy is notable. This discrepancy becomes even more intriguing when considering that initiating new or increasing sporting activities is often accompanied by concurrent positive changes in other health behaviors, such as improved dietary habits, which could increase or stabilize observed effects [32, 33].

Clearly, sporting activities are only one component of overall physical activity - particularly for individuals with obesity. Energy expenditure due to everyday physical activity (non-exercise activity thermogenesis), for instance, accounts for a far larger part of the total energy expenditure, and seems to play a more pronounced role for weight regulation in adult populations [11, 34]. Thus, the present results may be interpreted as an isolated and incremental effect of SPA.

### Strengths and limitations

A key strength of this study is its use of a large, population-based sample with repeated annual assessments, enhancing the representativeness and temporal resolution of our analyses. The ability to track individual changes in SPA and BMI over multiple years offers valuable insights into the dynamics of weight regulation.

Nonetheless, several limitations must be acknowledged. First, we analyzed the effects of energy expenditure due to self-reported SPA. Clearly, this may lead to an overestimation of physical activity or underestimation of actual BMI, which cannot be fully controlled. However, previous research suggests the overall validity of self-reported measures such as height and body mass [35, 36]. In contrast, the reliability and validity of self-reported SPA seems to be more uncertain [37]. Second, we did not control for confounds such as dietary intake, occupational physical activity, comorbidities, or psychosocial factors. Thus, while our results may yield representative estimates of the relation between MET and BMI for the subpopulations under consideration, we cannot unequivocally state their causal relation. Third, MET ratings are based on broad categories for each sport, which may not capture inter-individual intensity differences. As outlined above, assigning individuals who, for instance, perform high intensity running and who perform low intensity jogging identical MET values necessarily introduces unexplained variance to the model. However, while the random variation is certainly increased, we see no immediate reason that responses should introduce strong biases to our analyses. Fourth, not including the time spent on sporting activities in our measure of energy expenditure excludes an important theoretical component. However, because weighing MET with hours of sports per week had no incremental validity over the unweighted MET (as indicated by the correlational analyses), we prefer the more parsimonious measure and do not anticipate notable bias from this choice. Fifth, although the LISS panel is broadly representative of the Dutch population, we recognize that restricting the sample to individuals with multiple annual assessments may introduce some degree of selection bias – e.g. that more health-conscious individuals may be more likely to remain in the panel. Sixth, because SPA and BMI were assessed in different LISS questionnaires administered at different times of the year, there is a considerable delay between the two measures. While this likely introduces additional random variation compared to simultaneous assessment, our results should point to very stable and robust effects.

### Clinical implications and outlook

In current international guideline for obesity prevention and treatment, restriction of energy intake is considered as the most important component of multimodal treatment programs, with the strongest influence on body weight [5, 38]. Our main finding, that increases in SPA are related to only modest longitudinal BMI changes, are in line with the previous literature [39]. To support realistic goal setting, it is recommended to inform individuals with obesity about the limited success of exercise alone on weight reduction, while emphasizing the substantial health benefits (metabolic, cardiovascular, and psychosocial), which occur regardless of weight loss [5]. Especially in older individuals of our sample, effects on weight reduction are particularly small. However, for younger adults and individuals with obesity, SPA have a consistent small but notable impact on weight loss. Thus, early engagement of these individuals in higher-MET sports could leverage a more substantial impact on BMI, especially before weight gain imposes physical limitations on activity choices. In view of these differential effects depending on BMI and age group, clinicians may take our results into consideration when encouraging sporting activities based on a patient’s current BMI and mobility.

Future research should address whether combining dietary modifications and tailored exercise regimens yields synergistic effects on weight management across different BMI categories and age groups. Moreover, examining patterns of adherence and reasons for discontinuing specific types of sport could clarify how to sustain long-term lifestyle changes.

## Conclusions

In this representative longitudinal study, SPA measured via MET demonstrated clear cross-sectional and small but statistically significant longitudinal relationships with BMI. While individuals with higher BMI and younger age showed the greatest sensitivity to changes in SPA, overall weight trends remained relatively stable for those with lower BMI or older age. These findings underscore the nuanced role of SPA in weight management and the importance of individualized recommendations that consider age, weight status, and feasible exercise modalities.

While early and sustained engagement in SPA can help to optimize long-term weight outcomes and reduce obesity-related health risks, additional measures, such as healthy lifestyle practices, drugs or surgical therapy can be required for effective clinical interventions, if the BMI is already too high.

## Data Availability

The data are publicly available via https://en.centerdata.nl/liss-panel.

## Footnotes

1 This categorization was selected to achieve a balance between maintaining similar sample sizes across categories and ensuring homogenous differences between category thresholds.
